# Pes cavus and idiopathic scoliosis from school screening

**DOI:** 10.1186/1748-7161-8-S2-O6

**Published:** 2013-09-18

**Authors:** Hanene Belabbassi, Assia Haddouche, Abdelkader Ouadah, Houria Kaced

**Affiliations:** 1 Physical Medicine and Rehabilitation Department, University Hospital of Douera, Algiers, Algeria

## Background

The correlation between idiopathic scoliosis and cavus foot has previously been reported, and has been ascribed to possible lesions related to muscular imbalance influenced by the central nervous system.

This study assessed the rate of Pes cavus in scoliotic children. Two groups of patients were compared: those with idiopathic scoliosis and a control group without idiopathic scoliosis.

## Purpose

The aim of this study was to assess the rate of Pes cavus in children with and without scoliosis and to show if there is a significant difference between the two rates.

## Methods

A total of 82 children from a school screening program at the Physical Medicine and Rehabilitation Department in University Hospital of Douéra, Algiers were examined. Of those, 34 were boys and 48 were girls between the ages of 6 and 17 years. A number of measurements were assessed, including the trunk asymmetry in Standing Forward Bend followed by the Cobb angle in upright spine radiography.

We analyzed the footprints under weight bearing on the podoscope (mirror table). The first group included 43 children without scoliosis and the second group included 39 scoliotic children (Cobb angle ≥10 degrees). The presence of cavus foot (footprint type 1 and 2) in these two groups was examined. A statistical analysis was performed using the SPSS package.

## Results

Foot examination detected cavus foot in 13 (33.3%) out of the 39 scoliotics, including four (10.26%) typical and nine (23.08%) light cavus foot. Of the non-scoliotics, cavus foot was detected in 24 (55.8%) out of 43, with five (11.63%) typical and 19 (44.18 %) light cavus foot. There is a statistically significant difference between the Pes cavus rate in children without idiopathic scoliosis and those with idiopathic scoliosis. Comparing 55.8% to 33.3% we found Chi-square = 4.174 with P = 0.041.

## Conclusions and discussion

As reported elsewhere, our small sample noted the significant difference between Pes cavus in children with and without idiopathic scoliosis. In our study, the percentage of cavus foot was higher in healthy children than in patients with moderate scoliosis curves.
